# Ultrasonography of the Vagus Nerve in Parkinson’s Disease: Links to Clinical Profile and Autonomic Dysfunction

**DOI:** 10.3390/biomedicines13092070

**Published:** 2025-08-25

**Authors:** Ovidijus Laucius, Justinas Drūteika, Tadas Vanagas, Renata Balnytė, Andrius Radžiūnas, Antanas Vaitkus

**Affiliations:** 1 Department of Neurology, Medical Academy, Lithuanian University of Health Sciences, 44307 Kaunas, Lithuania; justinas.druteika@lsmu.lt (J.D.); renata.balnyte@lsmu.lt (R.B.); antanas.vaitkus@lsmu.lt (A.V.); 2 Department of Neurosurgery, Medical Academy, Lithuanian University of Health Sciences, 44307 Kaunas, Lithuania; andrius.radziunas@lsmu.lt

**Keywords:** vagus nerve, ultrasound, heart rate variability, Parkinson’s disease (PD), autonomic nervous system

## Abstract

**Background:** Parkinson’s disease (PD) is a progressive neurodegenerative disorder characterized by both motor and non-motor symptoms, including autonomic dysfunction. Structural alterations in the vagus nerve (VN) may contribute to PD pathophysiology, though existing data remain inconsistent. **Objective:** This study aimed to evaluate morphological changes in the VN using high-resolution ultrasound (USVN) and to investigate associations with autonomic symptoms, heart rate variability (HRV), and clinical characteristics in PD patients. **Methods:** A cross-sectional study was conducted involving 60 PD patients and 60 age- and sex-matched healthy controls. USVN was performed to assess VN cross-sectional area (CSA), echogenicity, and homogeneity bilaterally. Autonomic symptoms were measured using the Composite Autonomic Symptom Scale 31 (COMPASS-31). HRV parameters—SDNN, RMSSD, and pNN50—were obtained via 24 h Holter monitoring. Additional clinical data included Unified Parkinson’s Disease Rating Scale (UPDRS) scores, transcranial sonography findings, and third ventricle width. **Results:** PD patients showed significantly reduced VN CSA compared to controls (right: 1.90 ± 0.19 mm^2^ vs. 2.07 ± 0.18 mm^2^; left: 1.74 ± 0.21 mm^2^ vs. 1.87 ± 0.22 mm^2^; *p* < 0.001 and *p* < 0.02). Altered echogenicity and decreased homogeneity were also observed. Right VN CSA correlated with body weight, third ventricle size, and COMPASS-31 scores. Left VN CSA was associated with body size parameters and negatively correlated with RMSSD (*p* = 0.025, r = −0.21), indicating reduced vagal tone. **Conclusions:** USVN detects structural VN changes in PD, correlating with autonomic dysfunction. These findings support its potential as a non-invasive biomarker for early autonomic involvement in PD.

## 1. Introduction

Parkinson’s disease (PD), initially identified as “shaking palsy” by Dr. James Parkinson in 1817, represents a long-term, evolving condition marked by the degradation of both motor and non-motor functions. This disease profoundly affects individuals diagnosed with it, along with their families and those caring for them, by progressively impairing movement and muscle coordination [1].

PD incidence rate is usually comprised between 8 and 18 per 100,000 person-years [2]. The disease manifests in 1% of the population aged 60 years and above. Its occurrence is rare in individuals under 50 years of age, with prevalence increasing to 3% among the elderly people above 80. These findings underscore the age-dependent nature of the disease’s prevalence, with a substantial rise noted in advanced age groups [3]. PD is less frequent in Asia than in North America, Europe and Australia [4]. Environmental risk factors such as cigarette smoking, coffee and tea consumption, and exposure to pesticides, especially insecticides like organochlorines, have been associated with Parkinson’s disease (PD). These factors highlight the complex interplay between genetics and the environment in PD development [5]

PD is widely recognized as a disorder that affects motor skills but also profoundly impacts multiple systems including the autonomic nervous system [6]. These autonomic disturbances are often evident well before the motor symptoms manifest, highlighting the systemic nature of PD [7]. Non-motor features such as olfactory dysfunction, cognitive impairment, psychiatric symptoms, sleep disorders, autonomic dysfunction, pain, and fatigue are prevalent in the early stages of Parkinson’s disease leading to a decline in health-related quality of life [8].

Two fundamental pathological features characterize autonomic dysfunction in PD: autonomic neuronal degeneration and the accumulation of α-synuclein (αSyn). Neuronal degeneration manifests as the loss of neurons, degeneration of nerve fibers, and disruption of synaptic connections. The pathological accumulation of αSyn results in the formation of Lewy bodies, a hallmark of PD [9]. The central autonomic network, which includes the cerebral cortex, insular cortex, hypothalamus, brainstem, and spinal cord, is affected by both neuronal loss and αSyn deposition, as demonstrated in various neuropathological studies [10,11,12,13,14,15]. In the peripheral autonomic nervous system, structures such as VN, sympathetic nerve fibers, and enteric neural plexuses also exhibit significant neuronal loss and αSyn pathology. Notably, αSyn aggregation in these peripheral structures may occur earlier than in central nervous system regions, suggesting a potential peripheral origin of the disease process [15,16,17,18]. Moreover, aging induces structural degeneration in vagal afferents, impairing gut sensory feedback and motility control. Neurons in the nodose and dorsal root ganglia accumulate inclusions and lose Nissl substance, while their gastrointestinal terminals, including intraganglionic laminar endings (IGLEs) and villi afferents, undergo dystrophic changes such as axonal swellings, regression, and morphological distortions. These alterations likely result from reduced trophic support due to age-related loss of enteric neurons and interstitial cells of Cajal. The decline in vagal afferent integrity contributes to gastrointestinal dysfunctions like dysmotility and visceral hypersensitivity [19].

Early recognition of autonomic dysfunction is essential for effective intervention, as timely management can significantly improve patient outcomes. Treatment typically involves a multifaceted approach that begins with identifying and discontinuing medications that may contribute to or exacerbate autonomic symptoms. Patient education plays a crucial role, empowering individuals to recognize symptom triggers and adopt lifestyle modifications that can enhance autonomic stability. Non-pharmacological interventions, such as dietary modifications, physical therapy, compression garments, and fluid/salt intake adjustments, are often recommended as first-line strategies. In cases where symptoms persist or significantly impact quality of life, pharmacologic therapy tailored to the specific pathophysiological mechanisms involved may be necessary. A personalized, multidisciplinary approach incorporating neurologists, cardiologists, and autonomic specialists is often beneficial in optimizing patient care [20].

The vagus nerve (VN) has traditionally been studied for its efferent functions, particularly as the primary parasympathetic counterpart to the sympathetic nervous system, providing parasympathetic innervation to most organs. The parasympathetic and sympathetic nervous systems work in dynamic opposition, collectively regulating essential autonomic functions to maintain physiological balance [21]. Sympathetic activation causes blood vessel constriction, bronchodilation, an elevated heart rate, and the tightening of intestinal and urinary sphincters, reflecting its role in preparing the body for stress and heightened activity. In the neck, VN innervates most of the pharyngeal and laryngeal muscles, which are essential for swallowing and vocalization. It plays a crucial role in controlling speech and airway protection by regulating relevant muscles. In the thorax, VN provides the primary parasympathetic input to the heart, contributing to heart rate regulation by promoting a reduction in cardiac activity. This autonomic function helps maintain cardiovascular balance, particularly during periods of rest and digestion [22,23]. In PD autonomic dysfunctions such as orthostatic hypotension, which affects up to 58% of patients [24,25], and decreased heart rate variability, are common [26]. These issues are strongly associated with a worsening in the overall severity of the disease.

In this study we investigate structural changes in the VN in patients with PD and their potential association with clinical features of the disease. Using high-resolution ultrasound imaging, the study aims to evaluate the cross-sectional area, echogenicity, and homogeneity of the VN in PD patients compared to the control group. By analyzing these morphological parameters alongside clinical assessments, autonomic function tests, and neuroimaging findings, the study seeks to determine whether VN alterations can serve as an early biomarker of neurodegeneration in PD. Understanding these structural changes may contribute to improved diagnostic accuracy and the development of targeted interventions for autonomic dysfunction in PD.

## 2. Materials and Methods

The research adhered to the Declaration of Helsinki guidelines, and all participants provided informed consent. The protocol received approval from the Kaunas Regional Biomedical Research Ethics Committee, with the ethical clearance number BE-2-40 dated 17 May 2022. The study involved analyzing data from medical records and results from instrumental research of the participants.

The investigation was conducted at the Department of Neurology, Lithuanian University of Health Sciences, between 2022 and 2024. The study cohort comprised two groups: one group included 60 patients diagnosed with PD, and the other group consisted of 60 healthy controls, matched for age and sex with the PD patients. PD patients were adults, diagnosed according to widely recognized MDS Clinical Diagnostic criteria for PD [27]. Healthy individuals included in the study were also adults, who had no prior diagnoses of neurodegenerative diseases, polyneuropathies, neuromuscular junction disorders, endocrine disorders, cancer, or any other conditions that could influence the study results. We only excluded PD patients who did not sign the informed consent form.

All participants took part in an interview and completed questionnaires to collect clinical and demographic information, including age, sex, height, body mass index (BMI), hip and waist circumference, disease duration, comorbidities, and current medications. In patients with Parkinson’s disease, the VN was evaluated for cross-sectional area (CSA), homogeneity, and echogenicity. These morphological characteristics were analyzed in relation to clinical parameters, Sniffin’ Sticks Screening 12 test, Unified Parkinson’s disease rating scale (UPDRS), transcranial sonography (TCS) parameters, the Composite Autonomic Symptom Scale 31 (COMPASS-31) and heart rate variability (HRV) metrics. Vagus nerve ultrasonography (USVN) was performed for the control group patients as well.

### 2.1. Vagus Nerve Ultrasonography

Ultrasound evaluation of the vagus nerve was performed independently by two examiners using a Philips EPIQ 7 machine (Arbor Medical Corporation LT, Baltu pr. 145, Kaunas, LT-47125, Lithuania), with a high-resolution 4–18 MHz linear array transducer (CE 0086). The probe was oriented transversely in the lower cervical region, just above the clavicle, in line with the levator scapulae muscle, to visualize the nerve as it runs along the anterior scalene muscle. In B-mode imaging, the vagus nerve was recognized in close proximity to the carotid bifurcation, positioned posterior to the confluence of the internal and common carotid arteries, and displayed the characteristic sonographic appearance of a centrally hypoechoic structure encased by a hyperechoic connective tissue rim.

**Figure 1:**
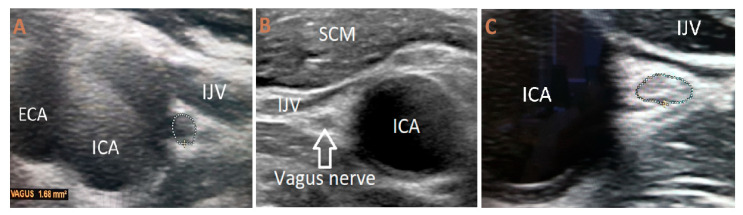
(**A**) Ultrasonography of the vagus nerve (USVN) showing a hypoechoic and homogeneous structure in a healthy volunteer. (**B**) USVN of a PD patient demonstrating an isoechoic and homogeneous appearance (**C**) USVN of a PD patient, demonstrating a hyperechoic and heterogenous appearance. The dotted circle indicates the cross-sectional area of the vagus nerve. SCM—sternocleidomastoid muscle; IJV—internal jugular vein; ICA—internal carotid artery; ECA—external carotid artery.

Measurements of the nerve’s diameter were obtained in millimeters from transverse images at two distinct sites: in the vicinity of the carotid bulb and at the level of the common carotid artery bifurcation. Both morphometric and qualitative characteristics were documented. The internal echotexture was described as either uniform or non-uniform, and echogenicity was categorized as hypoechoic, isoechoic, or hyperechoic (Figure 1).

For CSA measurements, a transverse imaging approach was used to delineate the hypoechoic nerve boundary against its hyperechoic margin, following the methodology described by Walter in 2018 [28]. Each examiner performed three independent measurements per side, ensuring a measurement precision of 0.01 mm^2^. The mean of the three values was calculated for each side, followed by an overall average. To minimize bias, both examiners conducted their assessments independently and were blinded to each other’s results.

### 2.2. Heart Rate Variability

Heart rate variability (HRV) refers to the variation in the time intervals between successive heartbeats and serves as a vital marker of neuro-cardiac function. It reflects the heart’s capacity to respond and adapt to both internal physiological changes and external environmental demands through modulation by the autonomic nervous system, highlighting the continuous interaction between the brain and the heart [29]. HRV is not only a reflection of cardiac function but also represents the dynamic interplay of multiple regulatory systems operating across various time scales. This adaptability is essential for maintaining homeostasis in the face of psychological and environmental challenges. Through autonomic regulation, HRV influences key physiological functions such as blood pressure control, respiratory dynamics, and vascular tone. Moreover, it extends its regulatory role to other bodily systems, including the gastrointestinal and musculoskeletal systems, illustrating the widespread impact of autonomic nervous system activity on overall health and function [30].

In this study, HRV was evaluated using data obtained from 24 h Holter monitoring in patients with PD. Three key HRV parameters were analyzed: SDNN (normal > 100 ms, borderline 50–100 ms, abnormal < 50 s), which represents the standard deviation of normal-to-normal (NN) intervals and serves as a general indicator of overall heart rate variability and cardiac risk; RMSSD (normal > 42 ms, abnormal < 20–25 ms), the root mean square of successive differences between NN intervals, which reflects parasympathetic (vagal) activity; and pNN50 (normal > 15%, abnormal < 3%), the percentage of successive NN intervals that differ by more than 50 milliseconds, also associated with parasympathetic nervous system function. Together, these metrics offer valuable insights into autonomic nervous system regulation.

### 2.3. COMPASS-31

The Composite Autonomic Symptom Scale 31 (COMPASS-31) is a standardized questionnaire used to screen and quantify symptoms of autonomic nervous system (ANS) dysfunction. Adapted from the original 169-item Autonomic Symptom Profile, it condenses the assessment into 31 targeted questions spanning six domains: orthostatic intolerance, vasomotor, secretomotor, gastrointestinal, bladder, and pupillomotor function. By providing a weighted score, it offers a quick yet comprehensive overview of autonomic symptom burden, facilitating both clinical evaluation and research applications. Total scores range from 0 to 100 with higher values indicating greater autonomic symptom burden. There is no strict cut-off value to indicate ANS dysfunction using COMPASS-31 and in the research field of PD, it is usually treated as a continuous variable [31].

### 2.4. Statistical Analysis

In this study, both descriptive and inferential statistical analyses were employed. Data processing was carried out using Microsoft Excel and IBM SPSS Statistics, version 29.0. For variables with a normal distribution, we applied a two-tailed *t*-test and presented results as mean values with corresponding standard deviations. In cases where the data did not meet the assumptions of normality, the Mann–Whitney U test was used, and results were reported as medians with minimum and maximum values. To evaluate correlations, the Pearson correlation coefficient was applied for normally distributed variables, while the Spearman rank correlation test was used for non-normally distributed data. A *p*-value of less than 0.05 was considered statistically significant for all analyses.

## 3. Results

### 3.1. Demographic and Clinical Characteristic of PD Patients

In a comparative study examining the demographic and clinical characteristics of PD and control group patients, each group consisted of 60 participants. The mean age in the PD group was 65.23 ± 9.22 years, compared to 63.62 ± 10.96 years in the control group, though this difference was not statistically significant (*p* = 0.123). The gender distribution (M:F ratio) showed a slight statistically insignificant variation (1.07:1 in controls vs. 1:1.31 in the PD group; *p* = 0.361). The hip circumference was found to be significantly higher in the PD group (*p* = 0.04). Other body measurements, including weight, height, waist circumference, and BMI, showed no significant differences, suggesting that PD does not substantially affect these characteristics compared to controls (Table 1).

PD group participants were included at any stage after visiting a neurologist (Table 2).

### 3.2. USVN Changes in PD Patients and Association with Clinical Features

In the control group, the average VN CSA measured 2.07 ± 0.18 mm^2^ on the right and 1.87 ± 0.22 mm^2^ on the left. Among patients with PD, the CSA was significantly smaller, averaging 1.90 ± 0.19 mm^2^ on the right (*p* < 0.001) and 1.74 ± 0.21 mm^2^ on the left (*p* < 0.02) when compared with controls (Table 3). When evaluating the relationship between VN CSA and PD stage, statistically significant associations were observed on both sides. On the right side, a significant correlation was found using Pearson’s correlation (*p* = 0.016), while on the left side, Spearman’s correlation revealed a similarly significant relationship (*p* = 0.003). However, when comparing CSA values across PD stages using the Kruskal–Wallis test, no statistically significant differences were identified between the groups. On the right side, the *p*-value was 0.066, and on the left side, 0.069, indicating trends toward significance but not meeting the conventional threshold (*p* < 0.05).

Significant differences in homogeneity and echogenicity of the VN were observed between the control and PD groups. The right VN was 96.7% homogenic in control group versus 23.3% in the PD group patients (*p* < 0.001). The left VN was 83.3% homogenic in control group versus 3.3% in PD group (*p* < 0.001). Hypoechogenicity was predominant in controls for both right (73.3%) and left VN (68.3%) compared to the PD group (16.7% and 15.0%, respectively), with isoechogenic and hyperechogenic patterns more prevalent in the PD group. Additional data are shown in Table 4.

Weight (*p* = 0.021, r = 0.296), third ventricle size (*p* = 0.026, r = 0.021), and COMPASS-31 score (*p* = 0.047, r = 0.257) showed significant correlations with the right VN measurements. Other clinical features, including age, gender, BMI, waist and hip circumference, and TCS measurements, were not significantly associated (*p* > 0.05). These findings suggest potential relevance of weight, third ventricle size, and COMPASS-31 score for the measurements of the right VN (Table 5).

Height (*p* = 0.002, r = 0.384) and weight (*p* = 0.003, r = 0.378) showed significant positive correlations with the left USVN, while third ventricle size (*p* = 0.007, r = 0.343) was also significantly associated. Other clinical features, including age, gender, BMI, waist and hip circumference, and TCS measurements, did not show significant correlations (*p* > 0.05) with the measurements of VN (Table 6).

No significant correlations were observed between the right USVN and HRV parameters, including SDNN (*p* = 0.667, r = −0.057), RMSSD (*p* = 0.289, r = −0.139), and pNN50 (*p* = 0.306, r = −0.134). In contrast, left USVN demonstrated a significant negative correlation with RMSSD (*p* = 0.025, r = −0.421), indicating a potential association between reduced vagal activity and left VN measurements. However, no significant correlations were found between the left USVN and SDNN (*p* = 0.930, r = 0.012) or pNN50 (*p* = 0.109, r = −0.209), as presented in Table 7.

## 4. Discussion

This study confirms and extends the findings of recent high-resolution ultrasound studies demonstrating significant atrophy of the VN in PD patients compared to controls [28,32,33,34,35,36,37]. Our results show a notable decrease in the CSA of the VN in PD patients, highlighting similar neurodegenerative mechanisms across different neurological disorders. Walter et al. reported a significant reduction in the VN caliber in PD patients which did not correlate with disease duration or severity, suggesting that vagal atrophy is a relatively early event in neurodegenerative processes [28]. Similarly, Pelz et al. found that both left and right VN were significantly thinner in PD patients, reinforcing the notion that neurodegenerative changes in the VN can be detected independently of the clinical stages of the disease [34]. However, not all studies demonstrate a significant reduction in VN size on ultrasound between groups [38,39]. Neither of the studies included body measurements, which could have influenced the outcomes. Furthermore, a previous study on healthy individuals demonstrated that BMI was significantly correlated with the size of VN, even between sides [40]. However, other studies suggest that height and weight [41] and other parameters as age, gender, height, weight, BMI, heart rate, systolic blood pressure, diastolic blood pressure in patients with diabetes mellitus do not correlate with the size of VN [42].

Our study reveals notable differences in echogenicity, particularly with the higher incidence of hypoechogenicity in the control group compared to the PD group. This observation suggests potential underlying differences in tissue architecture that could be indicative of neurodegenerative processes. Neuropathological research indicates that gastrointestinal dysfunction in PD is primarily linked to the accumulation of αSyn inclusions (Lewy neuritis) in both the enteric nervous system (ENS) and the dorsal motor nucleus of the VN. A few autopsy studies have been conducted, and both demonstrate structural and functional changes in various neurological conditions, including degenerative diseases such as PD. Research suggests that axonal degeneration, αSyn accumulation, and loss of parasympathetic innervation contribute to its deterioration [27]. Furthermore, axonal degeneration, along with dorsal motor nucleus degeneration, has been highlighted as a key factor in the development of non-motor symptoms (NMS) in PD [43,44,45]. Notably, still there appears to be a gap in existing literature regarding the specific role of echogenicity evaluation as a diagnostic marker in neurodegenerative diseases, which our findings begin to address.

The study on the correlation between USVN and clinical features in patients with PD provides valuable insights into potential diagnostic and prognostic indicators for the disease. The significant associations observed between right USVN measurements and clinical parameters such as weight, third ventricle size, and COMPASS-31 scores are particularly noteworthy. The left side USVN demonstrated significant associations between height, weight and third ventricle size. Enlargement of the third ventricle is linked to cognitive impairment not only in PD but also in other neurodegenerative disorders, including Alzheimer’s disease and multiple sclerosis [46,47,48,49], While it is not a specific indicator, its assessment using ultrasound adds value as an additional diagnostic tool.

The correlation between COMPASS-31 scores and ultrasound measurements of the right USVN in patients with PD. The analysis shows a weak positive correlation with an r-value of 0.257, which is statistically significant (*p* = 0.047). This suggests that there might be a relationship between the severity of autonomic symptoms and the anatomical features of the right VN in these patients. This finding highlights the potential importance of the VN in autonomic dysfunction in PD and warrants further investigation to explore this connection more deeply. However, some studies did not show a relationship between the VN CSA and autonomic neuropathy using COMPASS-31 [42].

The presented data also reveals interesting correlations between USVN measurements and HRV parameters in the PD group. Notably, the RMSSD parameter showed a significant negative correlation with left USVN measurements, suggesting a decrease in vagal tone and autonomic dysfunction in these patients. This correlation implies that structural changes in the VN, as reflected by ultrasound measurements, may influence HRV, potentially affecting autonomic regulation. Conversely, other HRV parameters like SDNN and pNN50 showed no significant correlation, highlighting the complexity of autonomic nervous system involvement in neurodegenerative diseases. This suggests that while some aspects of autonomic function (reflected by RMSSD) correlate with VN structure, other dimensions of HRV might be influenced by additional factors not captured solely by USVN.

Our study has several limitations. Firstly, the sample of our study is rather small and heterogenous. We gathered PD patients affected by the disease at various stages (as measured by Hoehn–Yahr stage) and using various PD medications that might influence HRV. The data regarding the influence of dopaminergic therapy for HRV is inconclusive. Some studies support the contributory effect of levodopa medication in early PD [50], while others report no significant change or even further reduction in HRV [51]. Systematic reviews and meta-analyses, including Heimrich et al. (2021), confirm that HRV is generally reduced in PD, reflecting impaired autonomic (particularly parasympathetic) control—but he emphasizes variations across disease stages and modalities [52]. One aim or our study was to grossly see if morphological changes in the VN could be related to any disruption of ANS and our study suggests that there are correlations but another study with more PD patients could answer the question if and how ANS is influenced by specific PD therapy. We also did not evaluate for specific comorbidities of PD patients that might influence HRV and other ANS functions.

## 5. Conclusions

The pronounced contrast in echogenic patterns between the control and PD groups could provide a foundation for using echogenicity as a non-invasive diagnostic tool. In our study, the control group exhibited more consistent hypoechogenicity, potentially representing a normal or less-altered state, whereas the variation observed in the PD group might reflect pathological changes, such as increased tissue density or gliosis.

Findings of this study highlight the relevance of ultrasonographical examination of the VN in clinical settings, particularly in the early diagnosis and monitoring of ALS. Continued research is necessary to further elucidate the mechanisms underlying these changes and to confirm whether these ultrasonographical markers can effectively predict disease progression or response to treatment.

## Figures and Tables

**Table 1:** Demographic and clinical characteristics of patients with PD and control group.

Variable	Control Group (SD = n)	PD Group (SD = n)	*p* Value ^a^
Number	60	60	
Age years, mean ± SD (range, years)	63.62 (SD = 10.96)	65.23 (SD = 9.22)	*p* = 0.123
Sex (M:F)	1.07:1	1:1.31	*p* = 0.361 ^b^
Weight kg, mean ± SD (range, kg)	80.47 (SD = 13.207)	79.07 (SD = 14.343)	*p* = 0.679
Height cm, mean ± SD (range, cm)	170.43 (SD = 9.553)	170.88 (SD = 9.152)	*p* = 0.905
Hip circumference ± SD (range, cm)	92.55 (SD = 9.722)	95.00 (SD = 12.337)	*p* = 0.04
Waist circumference ± SD (range, cm)	103.03 (SD = 7.811)	102.30 (SD = 8.850)	*p* = 0.504
BMI kg/m^2^ ± min/max (range, kg/m^2^)	26.93 (19.48–39.5)	27.68 (18.67–36.57)	*p* = 0.258 ^c^

^a^ Student’s *t*-test; ^b^ χ^2^ test for equality of proportions; ^c^ Mann–Whitney U test; BMI—Body Mass Index.

**Table 2:** Parkinson’s disease group patients by Hoehn–Yahr stages.

Hoehn–Yahr Stages	Number	M:F	Percentage (%)
1	3	3:0	5
1.5	3	0:3	5
2	6	3:3	10
2.5	5	4:1	8.3
3	18	12:6	30
4	14	7:7	23.3
5	11	2:9	18.3
	60	31:29	100

Hoehn–Yahr stages—system for describing how the symptoms of Parkinson’s disease progress.

**Table 3:** Cross-sectional area of the vagus nerve between groups.

Side of the VN	Control Group	PD Group	*p* Value
Right VN	2.07 ± 0.18	1.90 ± 0.19	<0.001
Left VN	1.87 ± 0.22	1.74 ± 0.21	<0.02

Values are stated using mean ± SD (standard deviation) values; χ^2^ test for equality of proportions.

**Table 4:** Homogeneity and echogenicity of the right and left VN between groups.

**A-1. Homogeneity of the Right VN Between Groups**			
**Homogeneity**	**Control Group**	**PD Group**	***p* Value**
Homogeneous N; %	58; 96.7%	14; 23.3%	<0.001
Heterogeneous N; %	2; 3.3%	46; 76.7%	
**A-2. Homogeneity of the left VN between groups.**			
**Homogeneity**	**Control Group**	**PD Group**	***p* Value**
Homogeneous N; %	50, 83.3%	2; 3.3%	<0.001
Heterogeneous N; %	10; 16.7%	58, 96.7%	
**B-1. Echogenicity of the Right VN Between Groups**			
**Echogenicity**	**Control Group**	**PD Group**	***p* Value**
Hypoechogenic N; %	44; 73.3%	10; 16.7%	<0.001
Isoechogenic N; %	16; 26.7%	38; 63.3%	
Hyperechogenic N; %	0	12; 20%	
**B-2. Echogenicity of the left VN Between Groups.**			
**Echogenicity**	**Control Group**	**PD Group**	***p* Value**
Hypoechogenic N; %	41; 68.3 %	9; 15%	<0.001
Isoechogenic N; %	19; 31.7%	39; 65%	
Hyperechogenic N; %	0	12; 20%	

Values are stated using absolute values and percentage values; χ^2^ test for equality of proportions. A-1, A-2—homogeneity of the vagus nerve between groups. B-1, B-2—echogenicity of the vagus nerve between groups.

**Table 5:** Correlations of the results of the right USVN and clinical features of PD patients.

Clinical Features	Mean (SD)	Right USVN	
		*p* Value	r Values
Age, years	65.23 (7.982)	*p* = 0.700	r = 0.510
Height, cm	170.88 (9.152)	*p* = 0.159	r = 0.184
Weight, kg	79.07 (14.343)	***p* = 0.021**	**r = 0.296**
BMI, kg/m^2^	26.93 (4.062)	*p* = 0.130 ^b^	r = 0.198
Waist circumference, cm	95.00 (12.337)	*p* = 0.145	r = 0.190
Hip circumference, cm	102.30 (8.850)	*p* = 0.807	r = 0.032
TKS right, cm	0.29 (0.073)	*p* = 0.197	r = −0.169
TKS left, cm	0.32 (0.082)	*p* = 0.651 ^b^	r = −0.060
Third ventricle	0.81 (0.149)	***p* = 0.026**	**r = 0.021**
Compass31	23.77 (13.458)	***p* = 0.047**	***p* = 0.257**
Gender	-	*p* = 0.271	*p* = −0.145

Values are stated using mean and standard deviation (SD) values; Pearson and Spearman (^b^) correlations. Statistically significant results (*p* < 0.05) are highlighted in bold.

**Table 6:** Correlations of the results of the left USVN and clinical features of PD patients.

Clinical Features	Mean (SD)	Left USVN	
		*p* Value	r Values
Age, years	65.23 (7.982)	*p* = 0.081	r = −0.227
Height, cm	170.88 (9.152)	***p* = 0.002**	**r = 0.384**
Weight, kg	79.07 (14.343)	***p* = 0.003**	**r = 0.378**
BMI, kg/m^2^	26.93 (4.062)	*p* = 0.112	r = 0.207
Waist circumference, cm	95.00 (12.337)	*p* = 0.065	r = 0.240
Hip circumference, cm	102.30 (8.850)	*p* = 0.556	r = −0.076
TKS right, cm	0.29 (0.073)	*p* = 0.180	r = −0.175
TKS left, cm	0.32 (0.082)	*p* = 0.674	r = −0.055
Third ventricle	0.81 (0.149)	***p* = 0.007**	**r = 0.343**
Compass31	23.77 (13.458)	*p* = 0.528	*p* = 0.830
Sex	-	*p* = 0.175	*p* = 0.210

Values are stated using mean and standard deviation (SD = n) values, Spearman correlations. Statistically significant results (*p* < 0.05) are highlighted in bold.

**Table 7:** Correlations between measurements of right VN and HRV parameters in PD group.

HRV Parameters	Mean (SD)	Right USVN	
		*p* Values	r Value
SDNN, ms	67.80 (SD = 19.422)	0.667	−0.057
RMSSD, ms	27.82 (SD = 11.967)	0.289	−0.139
pNN50, %	3.73 (SD = 2.736)	0.306 ^b^	−0.134
		**Left USVN**	
SDNN, ms	67.80 (SD = 19.422)	0.930	0.120
RMSSD, ms	27.82 (SD = 11.967)	**0.025**	**−0.421**
pNN50, %	3.73 (SD = 2.736)	0.109	−0.209

Values are stated using mean and standard deviation (SD = n) values; Pearson and Spearman (^b^) correlations. Statistically significant results (*p* < 0.05) are highlighted in bold.

## Data Availability

Data is contained within the article.
